# Balanced Thermal Insulation, Flame-Retardant and Mechanical Properties of PU Foam Constructed via Cost-Effective EG/APP/SA Ternary Synergistic Modification

**DOI:** 10.3390/polym16030330

**Published:** 2024-01-25

**Authors:** Hongfu Li, Longtao Hou, Yunpeng Liu, Zhiyu Yao, Lixing Liang, Dangxin Tian, Chunhui Liu, Junqiang Xue, Linshan Zhan, Yongqi Liu, Zhilu Zhen, Kangmin Niu

**Affiliations:** 1 School of Materials Science and Engineering, University of Science and Technology Beijing, Beijing 100083, China; 2 Hebei Construction Group Corporation Limited, Baoding 071051, China; 3 Hangzhou Hikvision Digital Technology Co., Ltd., Hangzhou 310052, China; 4 Microelectronics and Information Materials Research Center, Hangzhou Innovation Institute, Beihang University, Hangzhou 310053, China; 5 Hebei Province Prefabricated Building Technology Innovation Center, Baoding 071051, China

**Keywords:** rigid polyurethane foam, thermal insulation, flame-retardant, property balance, building wall material

## Abstract

To address the challenge of balancing the mechanical, thermal insulation, and flame-retardant properties of building insulation materials, this study presented a facile approach to modify the rigid polyurethane foam composites (RPUFs) via commercial expandable graphite (EG), ammonium polyphosphate (APP), and silica aerogel (SA). The resulting EG/APP/SA/RPUFs exhibited low thermal conductivity close to neat RPUF. However, the compressive strength of the 6EG/2APP/SA/RPUF increased by 49% along with achieving a V-0 flame retardant rating. The residual weight at 700 °C increased from 19.2 wt.% to 30.9 wt.%. Results from cone calorimetry test (CCT) revealed a 9.2% reduction in total heat release (THR) and a 17.5% decrease in total smoke production (TSP). The synergistic flame-retardant mechanism of APP/EG made significant contribution to the excellent flame retardant properties of EG/APP/SA/RPUFs. The addition of SA played a vital role in reducing thermal conductivity and enhancing mechanical performance, effectively compensating for the shortcomings of APP/EG. The cost-effective EG/APP/SA system demonstrates a positive ternary synergistic effect in achieving a balance in RPUFs properties. This study provides a novel strategy aimed at developing affordable building wall insulation material with enhanced safety features.

## 1. Introduction

In the face of the growing demand for building energy and the simultaneous concerns about the increasing greenhouse gas emissions, new building energy-saving insulation materials has become an important issue in the field of construction [1,2]. Lightweight-high-strength, low thermal conductivity, and high flame resistance are three essential evaluation criteria for external wall insulation materials [3,4]. For example, it is responsible for 40% of the total energy consumption in Europe [5], and good thermal insulation properties can provide a low carbon footprint for building energy consumption. Excellent flame retardant characteristics can effectively reduce property losses caused by fire incidents [6]. High mechanical performance can also reduce the occurrence of accidents such as falling wall cladding causing harm to individuals [7].

Currently, there is a wide variety of building insulation materials, such as polystyrene board, rock wool, polyurethane foam, vacuum insulation panels, gas filled panels, etc. [8,9]. Polystyrene boards have poor heat resistance, rock wool is prone to moisture absorption and layering risks, inorganic foam materials have high density and poor insulation performance, vacuum insulation panels are expensive and challenging in terms of construction and maintenance. In comparison, rigid polyurethane foam (RPUF) with its unique honeycomb closed-cell structure exhibits a lower thermal conductivity, lower density, and higher mechanical performance than other commercial insulation materials. It has been widely used as an insulation material in fields of pipes, household appliances, and cold chain transportation [3,10,11]. However, the flammability limits its widespread application as an exterior wall material in construction [11,12,13,14].

Different methods have been reported to improve the flammability of RPUF such as halogenated flame-retardants [15], phosphorous flame-retardants [16,17], graphite [18], aluminum hydroxide [19], expandable graphite (EG) [20], melamine [21], coating [22,23] and other additive flame-retardants [13,24]. However, a high loading amount is often needed in order to achieve satisfactory flame-retardant effects, which leads to a decrease in the mechanical properties of the RPUF and increases the risk of secondary damage caused by the detachment of the building external wall [25,26,27]. There is a significant synergistic flame-retardant effect where excessive amounts of EG can be effectively avoided through the use of EG in combination with phosphorus/nitrogen-based flame-retardants, while the resulting mechanical and thermal insulation properties are significantly reduced compared to neat RPUF [25,28,29,30,31,32,33,34]. How to achieve a balance among flame retardancy, thermal insulation, and mechanical properties of RPUF materials is a challenge and research hotspot in the field of external wall insulation material [35,36]. It would be ideal to introduce a material that has lower thermal conductivity than RPUF and can also act as a reinforcing effect. Therefore, silica aerogel (SA) has caught our attention.

SA is a new type of inorganic nano-porous material assembled from low-density 3D nanoparticles, which possesses excellent insulation performance and is an ideal functional insulation-modifying filler in the construction field [37,38,39,40]. However, the nano-surface effect and super-hydrophobic properties of SA can easily cause agglomeration, resulting in poor dispersion in polymers and leading to a significant increase in the viscosity of the polymer matrix, a decrease in the mechanical and bonding properties of the polymer material, and even a reduction in the insulation performance [18,38,41,42,43,44,45,46]. For example, Verdolotti et al. [42] prepared various RPUF materials modified with 1.5–7 wt.% of SA. As the SA content increased, the thermal conductivity of the samples gradually decreased from 30.88 mW/m·K to 24.19 mW/m·K. However, the compressive strength rapidly declined from 0.32 MPa to 0.03 MPa with an inability to maintain structural integrity. Fortunately, when we attempted to reduce the SA addition to approximately 1 wt.% [47], it not only resulted in good insulation effect but also can appropriately improve the mechanical properties of RPUF, achieving a balance between thermal insulation and mechanical properties. This provides valuable insights for proposing solutions to the aforementioned issues.

Herein, we proposed a ternary synergistic modified RPUF system incorporating EG/APP/SA, aimed at enhancing its flame-retardant properties without compromising the inherent insulation and mechanical properties of RPUF. Specifically, a control group of 1 wt.% SA modified RPUF was employed. Then, a flame-retardant system consisting of EG and APP were introduced to investigate the effects of different EG/APP ratios on the microstructure, compressive strength, thermal conductivity, thermal stability, and flame-retardant behaviors of the resulting EG/APP/SA/RPUF composite materials (EG/APP/SA/RPUFs).

## 2. Materials and Methods

### 2.1. Materials

Polyaryl polymethylene isocyanate (MDI) (PM200) (1.220–1.250 g/cm^3^, 150–250 mPa⋅s (RT), NCO content: 30.5–32.2%) and polyether polyol (HK-4110) (1.06 g/cm^3^, 3400 ± 400 mPa⋅s (RT), hydroxyl value of 430 ± 30 mg KOH/g) used for the preparation of RPUFs were purchased from Jining Huakai Resin Co., Ltd., Jining, China. Silicone oil (AK158) and dichlorofluoromethane (HCFC-141b) acted as surfactant and physical blowing agent, respectively, were also purchased from Jining Huakai Resin Co., Ltd., Jining, China. Triethylenediamine (A33) was used as catalyst provided by Jinan Jinbang Environmental Protection Technology Co., Ltd. Jinan, China. Deionized water was used as chemical blowing agent self-made in laboratory. Silica aerogel (SA) with particle size of 15 μm, thermal conductivity coefficient (RT) of 13.0 mW/mK, apparent density of 0.1 g/cm^3^ was supplied by Shenzhen Nano Technology Co., Ltd., Shenzhen, China. Expandabled graphite (EG) (E300) with particle size of 180 μm, expansion ratio of 300 mL/g, thermal conductivity coefficient (RT) of 290 mW/mK, apparent density of 0.54 g/cm^3^ and 99% purity was bought from Qingdao Yanhai Carbon Materials Co., Ltd., Qingdao, China. Ammonium polyphosphate of type II ((NH_4_PO_3_)N) (APP) with polymerization degree of 1000, particle size of 15 μm, phosphorus content of 31–32%, nitrogen conten of 14–15%, decomposition temperature of 280 °C, and 99.5% purity was available from Shouguang Puerchem Co., Ltd., Weifang, China. All the chemicals were used as received.

### 2.2. Preparation of EG/APP/SA/RPUFs

**Figure 1:**
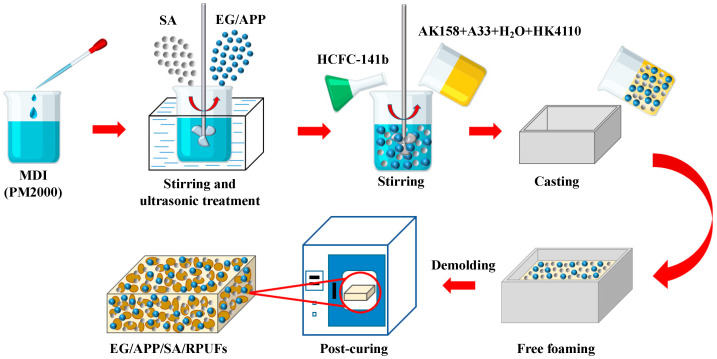
Preparation process for the EG/APP/SA modified RPUF samples.

Total seven control groups were designed for discussion. The formulation systems and abbreviated names of the samples are presented in Table 1 and Table 2, respectively. All PU foam samples were prepared using the free-rising method as illustrated in Figure 1 and described as follows. Specifically, the polyether polyol, SA powder, EG, and APP were dried at 80 °C overnight, followed by adding the rigid foam silicone oil, triethylenediamine, and distilled water sequentially to the polyether polyol. After the mixture was thoroughly stirred and blended to obtain the polyether polyol matrix, the SA and flame-retardants were added to the isocyanate matrix and mixed for 20–30 min in an ultrasonic water bath. Then the polyether polyol matrix was supplemented with dichlorofluoromethane and stirred for 30 s, followed by the isocyanate mixture was added to the polyether polyol blend and vigorously stirred for 8 s. The resulting reaction mixture was immediately poured into the mold for free-rising foam formation. After 30 min, the foam was demolded and placed in an oven for post-curing at 50 °C for 24 h. Finally, the flame-retardant modified SA/RPUF composite material of EG/APP/SA/RPUF was obtained. The preparation methods for neat RPUF and SA/RPUF are similar to this, and more details can be found in our previously work [47].

### 2.3. Characterization

#### 2.3.1. Density and Microstructure

At least five specimens of cubic shapes with side lengths of 50 mm were tested for each sample. The specimens were weighed using an electronic balance with an accuracy of 0.1% (g). The microstructure of the samples was observed using a scanning electron microscope (SEM) (JSM-6510A, Japan Electron Optics Laboratory Co., Ltd., Mitaka, Japan). Samples were cut into dimensions of 10 mm × 10 mm × 3 mm, and a thin layer of gold was sputtered onto the sample surface. The microstructural images were captured at an accelerating voltage of 20 kV. The particle size distribution of different materials was analyzed using Image J software v1.54.

#### 2.3.2. Chemical Structure

Fourier transform infrared spectroscopy (FTIR) (Nicolet iS 5 FT-IR, Thermo Fisher Technologies, Massachusetts, America) was performed in the wavelength range of 400–4000 cm⁻¹ using KBr pellet method. Prior to the experiments, the samples were placed in a drying oven at 50 °C for 24 h. Crystal phase analysis of the samples was carried out using a wide-angle X-ray diffractometer (WAXRD) (Ultima IV, Rigaku Corporation Global Website, Tokyo, Japan) in the scanning range of 5–60° with a step size of 0.05°. Elemental analysis of the sample residues after burning test was conducted using an energy dispersive spectrometer (EDS).

#### 2.3.3. Mechanical and Thermal Conductivity Properties

The compressive strength test specimens were cut into 100 mm × 100 mm × 50 mm dimension and compressed on an electronic universal testing machine at a rate of 5 mm/min until a relative deformation of 10% was reached. At least five samples were tested for each group. The thermal conductivity of samples was measured using an intelligent thermal conductivity tester (DRCD-3030, Tianjin Meister Test Machine Co., Ltd., Tianjin, China) according to the standard GB/T 10294-2008. Three samples with dimensions of 300 mm × 300 mm × 30 mm were performed and then averaged.

#### 2.3.4. Thermal Stability and Flame Retardancy

The thermal stability of the samples was evaluated using a thermogravimetric-differential scanning calorimetry (TG-DSC) analyzer (STA449F3, NETZSCH-Gerätebau GmbH, Selb, Germany) under a nitrogen atmosphere. The testing range was set from 25 °C to 700 °C, with a heating rate of 10 °C/min and a flow rate of 20 mL/min. By taking the first derivative of the thermogravimetric curve and plotting the resulting derivative thermogravimetry (DTG) curve, the temperature corresponding to each enthalpy change peak can be exactly determined. The limiting oxygen index (LOI) was determined using an oxygen index apparatus (XYC-100S, Chengde Xinma Test Instrument Co., Ltd., Chengde, China), with sample dimension of 150 mm × 10 mm × 10 mm. Each sample was tested at least 15 times to obtain reliable results. The vertical flame test (VFT) was conducted in accordance with ASTM D 3801-19 using a horizontal-vertical flame test apparatus (CZF-3, Chengde Xinma Test Instrument Co., Ltd., Chengde, China). The sample dimension for this test was 127 mm × 13 mm × 10 mm. Cone calorimeter test (CCT) (Vouch 6810, Suzhou Yangyi Volki Testing Technology Co., Ltd., Suzhou, China) was performed using a cone calorimeter instrument according to ISO 5660. The sample size was 100 mm × 100 mm × 25 mm. Each sample was performed under an external heat flux of 50 kW/m² to assess the fire performance and heat release. At least three samples were tested.

**Table 2:** The formulation and properties of different EG/APP/SA/RPUFs.

Samples	SA	EG	APP	RPUF	Formulation Density ^b^	Foam Density	Pore Fraction ^c^	Compressive Strength	Specific Strength	Thermal Conductivity Coefficient	T_5%_	T_50%_	T_max1_	T_max2_	Residues after TGA	Residues after CCT	LOI	UL-94
Units	wt.%	wt.%	wt.%	wt.%	kg/m^3^	kg/m^3^	vt.%	kPa	MPa/(g/cm^3^)	mW/mK	°C	°C	°C	°C	wt.%	wt.%	%	
SA					100					13					100			
EG					540					290					72.6			
APP					1740					/					34.9			
RPUF	0	0	0	100	1200 ^a^	45.6 ± 0.2	96.2	229 ± 12	5.02	24.4 ± 0.2	266.1	355.8	345.9	475.2	17.5	0.23	18.6	NR ^d^
SA/RPUF	1	0	0	99	1081	49.8 ± 0.2	95.4	396 ± 8	7.95	19.8 ± 0.2	267.7	358.2	346.4	476.8	19.2	0.24	18.3	NR
8EG/SA/RPUF	1	8	0	91	994	57.9 ± 0.6	94.2	268 ± 11	4.63	25.8 ± 0.1	266.5	357.0	343.8	458.8	22.3	7.24	24.8	V-1
8APP/SA/RPUF	1	0	8	91	1106	52.1 ± 0.8	95.3	348 ± 8	6.68	24.7 ± 0.2	249.8	332.4	314.3	478.7	23.4	4.35	24.4	V-1
2EG/6APP/SA/RPUF	1	2	6	91	1075	54.5 ± 0.7	94.9	431 ± 8	7.91	24.8 ± 0.1	257.4	330.8	298.9	474.2	25.1	4.92	25.8	V-0
4EG/4APP/SA/RPUF	1	4	4	91	1047	55.8 ± 0.4	94.7	394 ± 7	7.01	25.1 ± 0.2	258.5	336.7	309.0	473.1	26	6.06	25.6	V-0
6EG/2APP/SA/RPUF	1	6	2	91	1019	55.2 ± 0.6	94.6	342 ± 5	6.20	25.3 ± 0.2	283.8	370.6	321.6	476.3	30.9	6.85	26.1	V-0

Note: ^a^: ρ_RPUF_ was from the nominal density of polyurethane polymer material. ^b^: Formulation density = $\frac{100}{m_{SA}/\rho_{SA}+m_{EG}/\rho_{EG}+m_{APP}/\rho_{APP}+m_{RPUF}/\rho_{RPUF}}$. ^c^: Foam pore fraction = (1 − $\frac{Foam\;density}{Formulation\;density}$) × 100%. ^d^: “NR” means no rating.

## 3. Results and Discussion

### 3.1. Chemical Structure of EG/APP/SA/RPUFs

The FTIR and XRD results conducted on SA, EG, APP, neat RPUF, SA/RPUF, 8EG/SA/RPUF, and 8APP/SA/RPUF are presented in Figures S1 and S2. It can be observed, except for some new diffraction peaks corresponding to EG and APP observed in the XRD curves of 8EG/SA/RPUF and 8APP/SA/RPUF, respectively, no other significant changes in chemical structure are detected. The FTIR spectra of all RPUF samples also displays highly similar curves to that of neat RPUF. This indicates that the additives of EG, APP and SA are all primarily involved in physical interactions with RPUF during the foam preparation process.

### 3.2. Microstructure of EG/APP/SA/PRUFs

#### 3.2.1. Microstructure of SA, EG, and APP

The SEM micrographs and size distributions of the additives of SA, EG, and APP used for RPUF modification are presented in Figure S3. It is observed that SA exhibits an irregular particle structure, with the actual average particle size is smaller than the 15 μm data provided by the supplier. This can be attributed to the small size and high surface energy of the silica particles comprising SA, leading to significant aggregation and irregular shapes. In contrast, EG exhibits a plate-like structure, while APP exhibits a granular structure, both displaying particle sizes that are relatively close to the nominal values provided by the supplier.

#### 3.2.2. Microstructure of EG/APP/SA/PRUFs

The macroscopic morphologies of the various RPUF composite samples are shown in Figure S4. SA/RPUF and 8APP/SA/RPUF samples exhibit a similar appearance to neat RPUF, appearing milky white in color. However, the samples doped with EG appear gray in color. The overall structure integrity of all samples is good.

**Figure 2:**
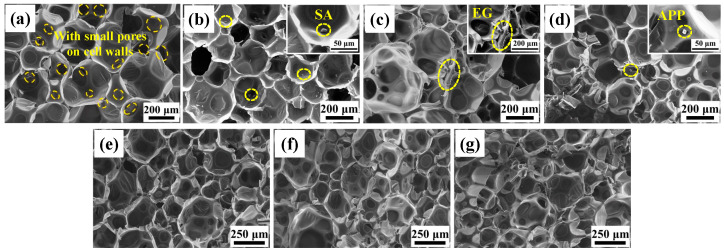
SEM images of (**a**) neat RPUF; (**b**) SA/RPUF; (**c**) 8EG/SA/RPUF; (**d**) 8APP/SA/RPUF; (**e**) 2EG/6APP/SA/RPUF; (**f**) 4EG/4APP/SA/RPUF; (**g**) 6EG/2APP/SA/RPUF.

The microscopic morphology observations of the various RPUF composite samples on the foam growth plane were conducted using SEM and shown in Figure 2. All RPUF samples exhibit closed honeycomb cellular structures with small pores on cell walls. The shape of the cells resembles a dodecahedron with a cross-section of pentagonal faces, and pillars and cell walls are formed at the boundaries between cells. The orientation of cell walls and pillars is random. This unique morphology of RPUF enhances heat transfer distance, resulting in excellent thermal insulation properties. In addition, functional particles such as SA, EG, and APP are dispersed in the cell or embedded within the cell walls in a predominantly physical manner observed from Figure 2b–d. This aligns with the results of FTIR and XRD in Section 3.1. Except for slightly smaller cell sizes in SA/RPUF, both neat RPUF and SA/RPUF exhibit well-defined honeycomb cellular structures and no obvious cell collapse is observed. However, the integrity and uniformity of cells in EG and APP modified RPUFs (Figure 2c–g) have been significantly compromised to varying degrees. The open cell ratio is also increased. Further, with increasing EG/APP ratio, the damage degree seems more pronounced, leading to larger cell sizes and broader cell size distributions.

**Figure 3:**
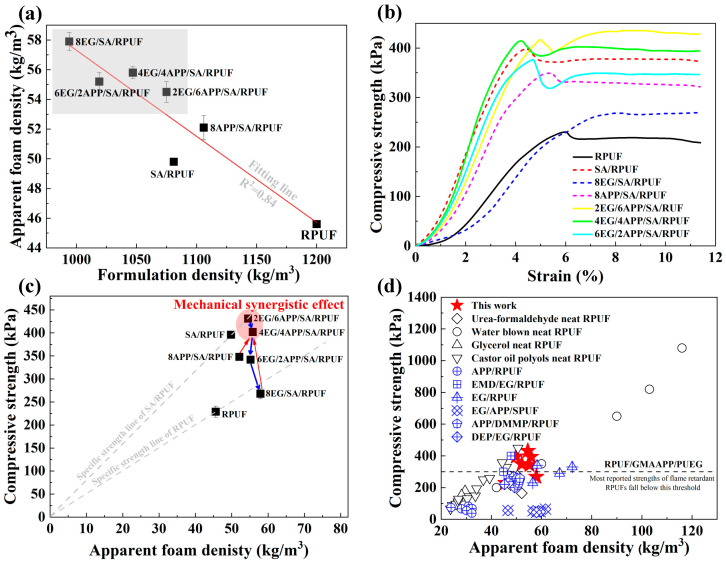
EG/APP/SA/RPUFs density and compressive properties of (**a**) relationship between formulation density vs apparent foam density; (**b**) compressive strength-strain curve; (**c**) specific compressive strength; (**d**) comparison with literature data: Urea-formaldehyde neat RPUF [13], Water blown neat RPUF [51], Glycerol neat RPUF [52], Castor oil polyols neat RPUF [53], APP/RPUF [26], EMD/EG/RPUF [29], EG/RPUF [49], EG/APP/SPUF [54], APP/DMMP/RPUF [55], DEP/EG/RPUF [56], and RPUF/GMAAPP/PUEG [25].

Based on the graphical analysis of the relationship between formulation density before foaming vs apparent foam density after foaming in Figure 3a, it is intriguing to observe that although the addition of low-density additives such as EG and SA is beneficial for reducing the initial formulation density, the apparent foam density actually increases after foaming. Furthermore, there exists a strong negative linear relationship between formulation density and apparent foam density. This can be attributed to the increased viscosity of the polymer matrix caused by the addition of additives such as SA, APP, and EG. The elevated viscosity, in turn, hinders the expansion of the RPUF foam and diminishes its foaming expansion ratio. Consequently, the pore fraction decreases (Table 2), leading to a higher apparent foam density. Among the three additives, EG exhibits the most prominent influence, as higher EG content results in lower pore fraction and higher apparent foam density as seen in Figure 3a gray area. On the other hand, the impact of SA and APP is comparatively less pronounced.

The reason for the effects of different additives on cell structure may be that the size of EG (180 μm) is comparable to the size of RPUF cells (approximately 150–400 μm), thus hindering the formation of complete cell structures. This leads to the convergence of multiple incomplete small cells, resulting in an apparent increase in cell size. Furthermore, under equal mass fraction conditions with APP, the lower density characteristics of EG (540 kg/m^3^ given in Table 2), which result in a larger fill volume, make it more prone to increasing the viscosity of the polymer matrix and causing more severe difficulties in cell growing and achieving uniform dispersion, which also adversely affects the cell structure.

While the addition of inorganic additives such as APP and SA can also introduce viscosity increase and agglomeration issues, the smaller particle size (approximately 15 μm) of these additives can act as heterogeneous nucleating agents during foaming [48]. This effectively limits the increase in cell size but is more favorable for maintaining the structural integrity. Consequently, it results in a more uniform and smaller cell structure compared to EG samples.

In conclusion, the SEM results suggest that the addition of larger-sized EG has a dual impact on foam formation: reducing both foaming expansion rate and cell integrity. This finding provides a basis for understanding the subsequent discussion on the influence of compressive strength.

### 3.3. Compressive Properties of EG/APP/SA/RPUFs

The typical compressive stress-strain curves of various RPUF composite samples are shown in Figure 3b. Overall, the addition of SA, EG, and APP has improved the mechanical properties of RPUF. This indicates that the appropriate inclusion of inorganic additives has an overall strengthening effect despite having some adverse effects on the cell formation during foaming process. Especially in the case of SA/RPUF, as consistent with the introduction argument, the addition of 1 wt.% SA alone results in a 73% improvement in the compressive strength compared to pure RPUF, increasing it from 229 kPa to 396 kPa. The possible reason is that SA has an ultra-low density (100 kg/m^3^ given in Table 2), and even with just a 1wt.% weight fraction, it exhibits a high reinforcement filling volume rate. This forms a crucial foundation for the design and implementation of EG/APP/SA/RPUFs in this study, aiming to achieve a balanced performance in thermal insulation, flame retardancy, and mechanics.

In addition to the properties of the material itself, density is also an important parameter that influences the mechanical properties of foam structures [49,50]. So, the specific compressive strength (foam compressive strength divided by apparent foam density) is further analyzed in Figure 3c. Data from this work and literatures of various RPUF samples [13,26,29,49,51,52,53,54,55,56] are also presented in Figure 3d. It can be clearly observed that the compressive strength of all PU foam samples exhibits a dependence of linear with respect to the foam density. That is to say, density is the direct factor influencing foam strength regardless of its composition.

From a more detailed analysis of apparent strength and specific strength in Figure 3b,c, it can be furtherly revealed: (1) except for the 8EG/SA/RPUF sample, all other samples exhibited higher specific strength compared to neat RPUF of 5.02 MPa/(g/cm^3^), respectively. This indicates that these samples demonstrated effective particle dispersion strengthening, surpassing the strength improvement achieved solely through increased foam density of neat RPUF itself. (2) For samples containing EG and (or) APP, both apparent strength and specific strength are higher when EG and APP are used together compared to when EG or APP is added alone (red arrows in Figure 3c). This demonstrates a noticeable synergistic enhancement effect. Particularly, when the EG:APP ratio is 2:6 and 4:4, the strength even surpasses that of SA/RPUF. However, this synergistic enhancement effect shows a rapid decrease as the ratio of EG/APP increases until the specific strength of 8EG/SA/RPUF falls below that of RPUF (blue arrows in Figure 3c). This can be explained by the changes in the SEM microscopic cell structure as discussed in the previous section. Specifically, larger EG particles, especially at higher concentrations, can cause severe damage to the PU cell structure, affecting the formation of a continuous support pathway composed of foam cell walls that actually bear the load, finally resulting in the loss of the stiffening and strengthening effect. However, when a fixed addition proportion is used, the inclusion of a small amount of APP can effectively reduce the volume filling amount of EG, thereby mitigating the damage caused to the PU foam cells. On the other hand, smaller-sized APP can additionally contribute to a more efficient dispersion strengthening mechanism by being distributed between EG and within the foam cell walls. Actually, most of the mechanical properties in this study are significantly higher than those reported in literature for flame-retardant modified RPUF composites (blue marks) as indicated in Figure 3d. This also demonstrates the synergistic enhancement effect of EG/APP/SA on RPUF.

### 3.4. Thermal Conductivity Behaviors of EG/APP/SA/PRUFs

**Figure 4:**
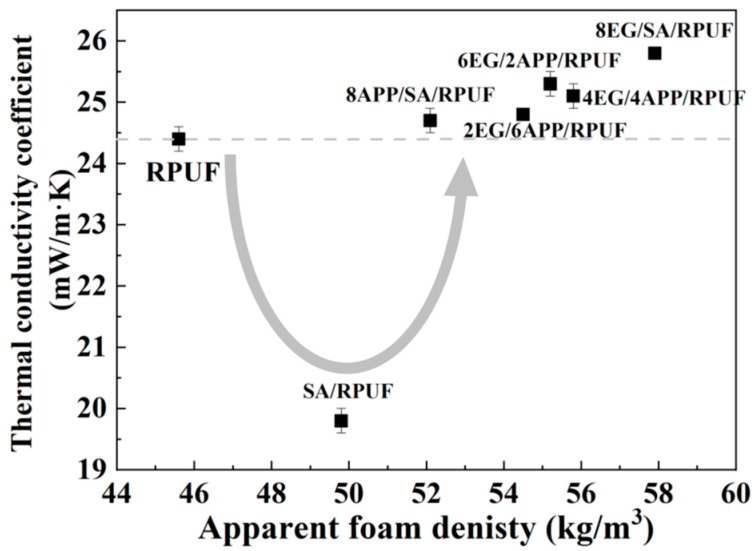
Thermal conductivity behaviors of EG/APP/SA/RPUFs.

As shown in Figure 4 and Table 2, the addition of 1 wt.% SA to the RPUF matrix results in a further reduction of its thermal conductivity from 24.4 mW/mK to 19.8 mW/mK, achieving a decrease of 18.9%. The mechanism may be similar to that of the mechanical property reinforcement caused by the high filling volume effect resulting from ultra-low density of SA.

Based on the SA/RPUF samples, the addition of EG and APP individually in the 8EG/SA/RPUF and 8APP/SA/RPUF samples increased the thermal conductivity from 19.8 mW/mK to 25.8 mW/mK and 24.7 mW/mK, respectively, restoring it to the level of neat RPUF. This is because inorganic materials such as EG and APP generally have higher thermal conductivities than PU polymers and their presence in the foam cell walls, either internally or on the surface, increases the thermal conductivity of the solid-phase heat transfer channels [50]. Additionally, the increased viscosity of the polymer mixture resulted in increases in density and open cells ratio, which also contributes to the increase in the thermal conductivity coefficient [57].

In the case of the EG/APP/SA/RPUFs, the thermal conductivity falls between that of 8APP/SA/RPUF and 8EG/SA/RPUF, and increasing with the ratio of EG/APP as shown in Figure 4. This is attributed to the higher thermal conductivity of EG compared to APP and the compromised foam cell structure integrity of RPUF. Furthermore, the thermal conductivity is influenced by phonon transfer. When a higher proportion of flame-retardant is used, the aggregation increases, promoting phonon transfer and consequently increasing the thermal conductivity [58]. However, due to the significant contribution of the SA/RPUF control group in reducing thermal conductivity, the increase in thermal conductivity of the EG/APP/SA ternary modified RPUF composite is not significant compared to neat RPUF. Actually, the thermal conductivities obtained in this study are significantly lower compared to those reported in most literature for flame-retardant modified RPUF composites [49,50,56,59,60,61,62,63]. This indicates that the EG/APP/SA combination modification of RPUF demonstrates a promising potential for achieving a balance between thermal insulation and flame retardancy.

### 3.5. Thermal Stability of EG/APP/SA/PRUFs

**Figure 5:**
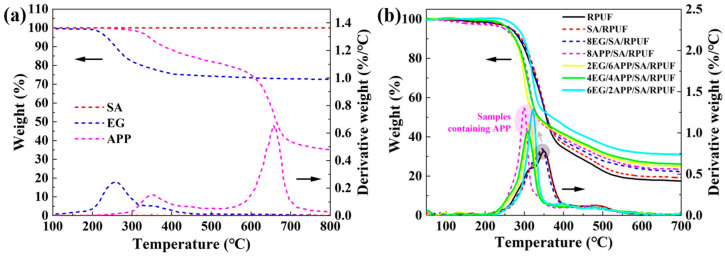
TGA and DTG curves for (**a**) SA, EG and APP additives; (**b**) EG/APP/SA/RPUFs. (The arrow direction indicates the corresponding Y-axis of the curves.)

According to the TGA results shown in Figure 5a and Table 2, it can be observed that SA is a highly stable material. The mass of SA shows no significant change or decomposition as the temperature rises to 800 °C. On the other hand, EG starts to rapidly decompose around 200 °C, and its residual mass at 800 °C is 72.6 wt.%. The decomposition of APP occurs in two stages, with the corresponding fastest decomposition rates observed at temperatures of 353 °C and 658 °C. The first stage of mass loss from 300 °C to 400 °C is primarily attributed to the release of NH_3_, H_2_O and the formation of poly(phosphoric acid). While the second stage from 600 °C to 700 °C corresponds to a mass loss of approximately 45%, which is associated with the release of P_2_O_5_ by further thermal degradation [64]. The residual mass of APP at 800 °C is 34.9 wt.%.

The relative mass loss of EG/APP/SA/PRUFs with temperature variation is shown in Figure 5b. Although EG and APP exhibit significant mass loss behavior in the low-temperature range of 200–400 °C, their low content and high residual mass characteristics make them not prominently displayed in the thermal degradation curves of the RPUF composites. The dominant feature of the EG/APP/SA/PRUFs curves is the degradation behavior of PU itself. Specifically, all RPUF composite materials exhibit two degradation processes: a first-stage degradation with mass reduction in the temperature range of 200–380 °C, which may be attributed to the degradation of the hard segments of the RPUF polymer chains, and a second-stage degradation in the temperature range of 380–600 °C, which is associated with the oxidative decomposition of the polyisocyanate and aromatic compounds in the soft segments of the RPUF polymer chains [65,66].

Compared to neat RPUF, the TGA curves of the SA/RPUF and 8EG/SA/RPUF samples almost overlap with RPUF due to the excellent high-temperature stability of SA and EG. This indicates that SA and EG, primarily through physical interactions, do not significantly alter the thermal degradation mechanism of RPUF. However, for all composite foam samples containing APP (indicated by the pink area in Figure 5b), the maximum weight loss temperature (T_max1_) is reduced compared to neat RPUF (indicated by the gray area in Figure 5b), and the reduction becomes more significant with increasing APP content. This is attributed to the promotion of PU polymer chain degradation catalyzed by the poly(phosphoric acid) generated during the first stage thermal decomposition of APP [67]. However, it is also due to this decomposition process that the subsequent interaction between poly(phosphoric acid) and polyhydric alcohol (degradation product of PU) takes place, resulting in P-O-P and P-O-C cross-linked protective layers on the foam and loosely distributed worm-like EG surface and enhancing the thermal stability and flame retardancy of the remaining RPUF [30]. As a result, this synergistic mechanism ensures that the residual mass of the EG/APP composite (solid line in Figure 5b) surpasses that of the individual components (dotted line in Figure 5b) when used independently. Particularly, the 6EG/2APP/SA/RPUF sample achieves a residual mass of 30.9 wt.%, which represents a 77% increase compared to the 17.5 wt.% of the neat RPUF. The initial degradation temperature T_5%_ and the 50% mass loss temperature T_50%_ have also been correspondingly increased.

### 3.6. Flame-Retardant Properties of EG/APP/SA/PRUFs

#### 3.6.1. Flammability Behaviors

**Figure 6:**
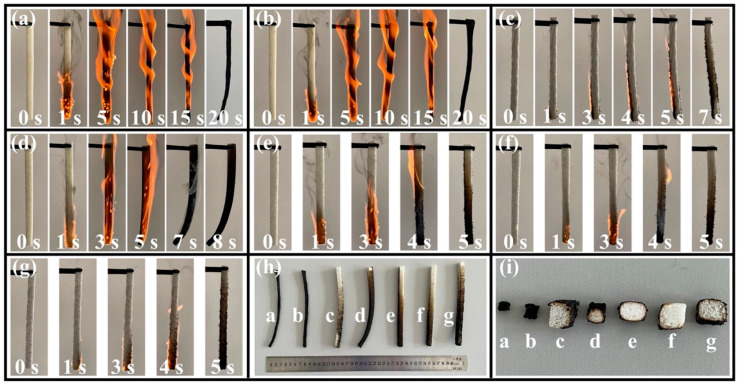
Digital photographs of VFT for samples of (**a**) neat RPUF; (**b**) SA/RPUF; (**c**) 8EG/SA/RPUF; (**d**) 8APP/SA/RPUF; (**e**) 2EG/6APP/SA/RPUF; (**f**) 4EG/4APP/SA/RPUF; (**g**) 6EG/2APP/SA/RPUF; (**h**) the external appearance; (**i**) the cross-section of the samples after VFT.

The LOI and VFT results are shown in Table 2 and Figure 6. The flammability of polymers is usually evaluated by the time to ignition (TTI) and self-extinguish time. As control groups, the neat RPUF and SA/RPUF samples exhibit LOI values of only 18.6% and 18.3%, respectively. In the VFT, they are quickly ignited and engulfed by the flame within a few seconds (Figure 6a,b), indicating that they do not meet the UL-94 fire rating requirements. However, upon the addition of 8 wt.% EG or APP individually, the LOI of 8EG/SA/RPUF and 8APP/SA/RPUF increases to 24.8% and 24.4%, respectively. In the VFT, they self-extinguish after 7 s and 8 s of ignition, respectively, achieving a flame retardancy rating of V-1 according to the UL-94.

Upon simultaneous addition of EG and APP, the LOI of 2EG/6APP/SA/RPUF, 4EG/4APP/SA/RPUF, and 6EG/2APP/SA/RPUF increases to 25.8%, 25.6%, and 26.1%, respectively. Furthermore, all three groups of samples achieve the V-0 fire rating, indicating self-extinguishing within 5 s after flame removal. Additionally, as observed from the burned sample morphology in Figure 6h,i, the samples with EG and APP additives form a self-extinguishing char layer on the surface, while the internal substrate remains almost unchanged in color and maintains its original shape and integrity. In contrast, the neat RPUF and SA/RPUF only leave char residues with significant volume shrinkage. In summary, the addition of either EG or APP individually can enhance the thermal stability of the RPUF composite materials at high temperatures. However, the synergistic effect of adding both EG and APP together is even more significant.

#### 3.6.2. Fire Behaviors

**Figure 7:**
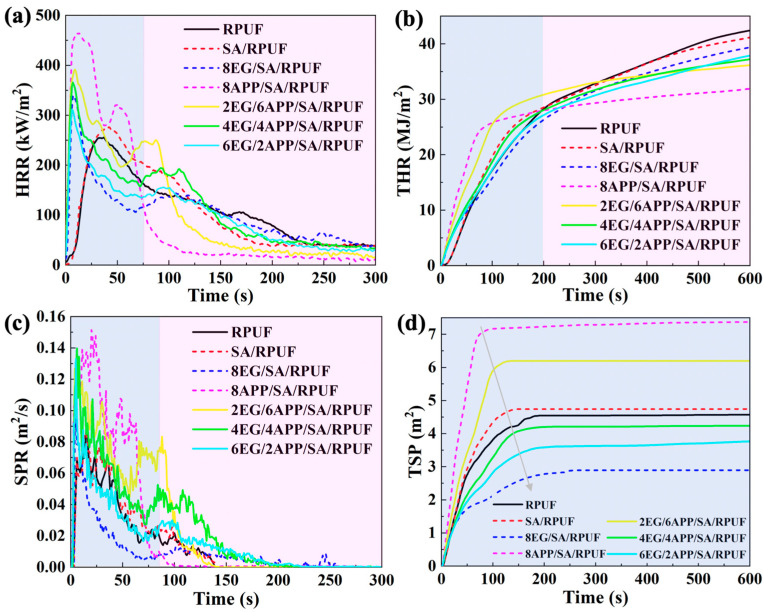
CCT curves of EG/APP/SA/RPUFs for (**a**) HRR; (**b**) THR; (**c**) SPR; (**d**) TSP. (Blue background: EG predominance-area, Pink background: APP predominance-area).

Heat and smoke are two major hazards of fires. In this study, the heat release behaviors of RPUFs are described by heat release rate (HRR) and total heat release (THR). The smoke emission behaviors of RPUFs are described by smoke production rate (SPR) and total smoke production (TSP). The CCT results are depicted in Figure 7 and summarized in Table 3.

In terms of heat release performance, similar to the aforementioned VFT results, neat RPUF and SA/RPUF composite materials exhibit nearly identical values in HRR, TTI, peak heat release rate (PHRR), THR, TSP, and mass of residual char (MRC). The two curves also closely overlap. This outcome is attributed to the fact that a small amount of SA can only form a thin protective layer on the surface of RPUF, which does not effectively reduce the heat transfer rate during combustion [68]. However, for the flame-retardant modified SA/RPUF composites incorporating EG or APP separately, the TTI for 8EG/SA/RPUF and 8APP/SA/RPUF is extended from 2 s to 7 s and 5 s, respectively, while the PHRR increases to 340 kW/m^2^ and 464 kW/m^2^ from 276 kW/m^2^ (Figure 7a), respectively. Combining the previous TGA results in Figure 5, this can be attributed to the thermal decomposition of intercalation agents within EG during heating, resulting in the generation of a significant amount of expansion heat. While APP rapidly decomposes and generates gas upon heating, disrupting the surface carbon layer of the matrix, leading to a higher HRR. However, precisely due to these characteristics of EG and APP, the remaining portion of RPUFs forms a protective layer on the surface due to the pre-degraded components, resulting in a reduced THR (compared to 42.3 MJ/m^2^ of SA/RPUF, the THR for 8EG/SA/RPUF and 8APP/SA/RPUF decreases by 5.7% and 24.2%, respectively), providing better fire safety for the materials [67]. Furthermore, it can also be observed that although samples containing APP exhibit higher HRR and THR in the initial 70–200 s, they show lower HRR and THR thereafter until CCT is over, and this feature becomes more pronounced with increasing APP content in EG/APP/SA/RPUFs (Figure 7a,b). This indicates that APP is more effective in reducing the THR of the RPUF composite compared to EG. On the other hand, EG can compensate for the heat release behavior of APP in the low-temperature stage.

In terms of smoke performance, it can be observed from Figure 7c,d and Table 3 that the TSP of neat RPUF and SA/RPUF is 4.58 m^2^ and 4.74 m^2^ respectively, showing minimal change. However, for 8EG/SA/RPUF and 8APP/SA/RPUF, the TSP is 2.89 m^2^ and 7.37 m^2^ respectively. This indicates that EG is efficient in reducing the TSP of RPUF composites, while APP, on the contrary, significantly increases the smoke production of RPUF. Moreover, a higher APP content leads to higher smoke production. As the proportion of EG/APP surpasses 4:4, the TSP of EG/APP/SA/RPUFs drops below that of neat RPUF.

The residual char yields (Table 3) for neat RPUF and SA/RPUF at the end of combustion are only 0.23 wt.% and 0.24 wt.%, respectively. For the 8EG/SA/RPUF and 8APP/SA/RPUF samples, the residual char yields increase to 7.24 wt.% and 4.35 wt.%, respectively. With the combined addition of EG and APP, the char yields for 2EG/6APP/SA/RPUF, 4EG/4APP/SA/RPUF, and 6EG/2APP/SA/RPUF further increase to 4.92 wt.%, 6.06 wt.%, and 6.85 wt.%, respectively.

The above results demonstrate that the EG/APP/SA/RPUF system can yield a favorable synergistic flame-retardant effect in terms of heat-smoke-residual char, thereby reducing the fire risk effectively.

#### 3.6.3. Residual Char Morphology and Elemental Composition

**Figure 8:**
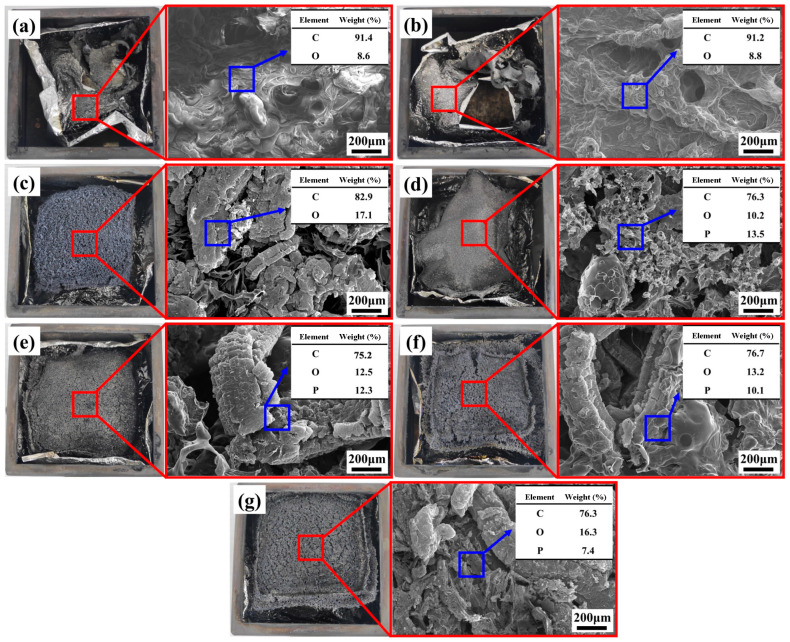
Digital and SEM images after CCT: (**a**) neat RPUF; (**b**) SA/RPUF; (**c**) 8EG/SA/RPUF; (**d**) 8APP/SA/RPUF; (**e**) 2EG/6APP/SA/RPUF; (**f**) 4EG/4APP/SA/RPUF; (**g**) 6EG/2APP/SA/RPUF.

To further elucidate the flame-retardant mechanisms of SA, EG, and APP during the combustion of RPUF composite materials, the morphology and elemental composition of the residual char after CCT were analyzed in Figure 8. It can be observed that pure RPUF and SA/RPUF samples show only a small amount of irregular layered residue after combustion, with numerous microstructural cracks. Oxygen and heat can directly penetrate through these surface cracks, resulting in ineffective combustion suppression. In contrast, due to the thermal expansion of EG, the surface of 8EG/SA/RPUF is covered by larger-sized “worm-like” layered graphite, creating a thick but porous residual char that exhibits a typical “popcorn effect” [69]. However, the interface strength between EG and the RPUF matrix is relatively weak. In comparison, during combustion in Figure 8d, APP acts as both an acid source and a foaming agent, promoting dehydration and cross-linking protective layer on the RPUF matrix. This leads to a denser char layer with increased viscosity, contributing to enhanced interfacial strength. However, the char layer is thinner, resulting in a less effective barrier.

When EG and APP are combined (Figure 8e–g), the smaller products generated from APP decomposition fill the gaps between the worm-like EG structures, resulting in a more compact and continuous char layer for the RPUF material. The matrix shape is preserved, preventing the formation of shrinkage defects, thereby effectively achieving heat and oxygen isolation. In this study, the sample 6EG/2APP/SA/RPUF presents the densest residual char structure, corresponding to the optimal TGA residues, CCT residues, LOI, and V-0 flame-retardant level.

Through EDS spectrum, it can be revealed that the residual char of neat RPUF and SA/RPUF are primarily composed of carbon and oxygen elements, with a higher proportion of carbon and a lower proportion of oxygen. However, with the addition of EG in 8EG/SA/RPUF, incomplete combustion leads to an increase in the mass percentage of oxygen and a decrease in the mass percentage of carbon in the residual char. 8APP/SA/RPUF shows the presence of phosphorus in addition to oxygen and carbon, but nitrogen is not detected. This confirms that during combustion, APP releases non-combustible nitrogen-containing gases such as NH_3_. When both EG and APP are introduced in EG/APP/SA/RPUFs, the residual char displays higher contents of both oxygen and phosphorus, indicating a notable synergistic flame-retardant effect.

#### 3.6.4. Flame Retardant Mechanism for EG/APP/SA/RPUFs

**Figure 9:**
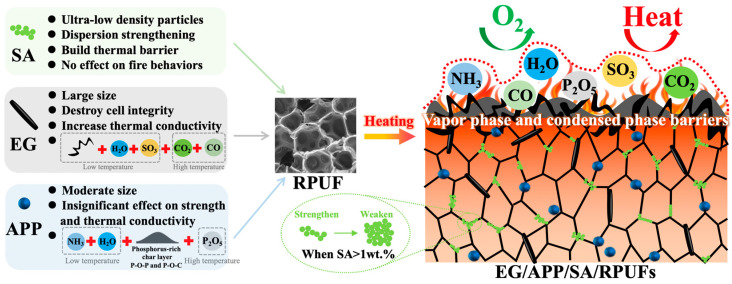
Schematic diagram of flame retardant mechanism for ternary synergistic modification EG/APP/SA/RPUFs.

Based on the preceding TGA, LOI, VFT, CCT results, the synergistic flame-retardant mechanism of EG/APP/SA/RPUFs can be inferred as follows and depicted in Figure 9. When a heat source is applied to the EG/APP/SA/RPUFs, combustible gas is released from the closed foam cells and rapidly ignited. Simultaneously, EG flakes and APP particles located within the cell absorb heat energy. The synergistic flame-retardant action initiates with the endothermic expansion of EG at 200 °C (as seen in Figure 5a). Intercalation agent (the main component in E300 EG is sulfuric acid) existing between graphite layers undergo instantaneous evaporation and decomposition accompanied by SO_3_ and H_2_O, generating significant expansion heat. This results in the formation of a thick and porous “worm-like” carbonized layer along the axial direction. This carbonized layer can maintain stability at high temperatures up to nearly 800 °C, hindering direct contact between the heat source, oxygen, and the matrix. Subsequently, as the temperature rises to 300 °C, APP begins to decompose and release NH_3_, which can effectively act as non-combustible gas blocking the supply of oxygen. The decomposition of APP simultaneously generates inorganic acid like poly(phosphoric acid), leading to the formation of P-O-P crosslinking protective carbide layer due to dehydration between poly(phosphoric acid) [70,71]. Meanwhile, poly(phosphoric acid) catalyzes the degradation of PU to produce polyhydric alcohol and undergoes esterification reaction with it, completing the second dehydration reaction to form P-O-C cross-linking structures. This is also why EG/APP/SA/RPPUs exhibit the highest rate of weight loss around 300 °C accompanied by highest HRR and SPR as shown in Figure 5b and Figure 7, respectively.

Thanks to the non-combustible gases of NH_3_, SO_3_, and H_2_O produced by the synergistic flame-retardant system, a thick and dense hybrid carbon barrier protective layer composed of expandable graphite/P-O-P/P-O-C cross-linked networks, results in the outstanding flame-retardant performance of EG/APP/SA/RPUFs at temperatures below 700–800 °C in terms of smoke, heat, self-extinguishing, and residual weight, etc. If the temperature continues to rise above 700 °C, it may lead to further thermal decomposition of the P-O-P and P-O-C structures, resulting in the production of P_2_O_5_ and the combustion of graphite to generate CO_2_ and CO gases [30,64].

It is worth noting that, due to the larger particle size and higher thermal conductivity of EG, excessive addition of EG will result in a decrease in the mechanical strength of the RPUF matrix and an increase in thermal conductivity. Furthermore, because APP thermally decomposes to produce a large amount of gases such as CO_2_, NH_3_, H_2_O, and P_2_O_5_ [72,73], it can disrupt the formation of the protective char layer on the matrix surface, leading to an increase in total smoke production (as shown in Figure 7d). Therefore, it is essential to control the proportion of EG and APP additives effectively to achieve a balanced performance in terms of mechanics/insulation/flame-retardancy in EG/APP/SA/RPUFs. 

### 3.7. Ternary Synergistic Mechanism of EG/APP/SA in RPUF

Taking into account the comprehensive assessment of various physical, chemical, mechanical, insulation, oxygen index, and smoke-heat related outcomes, the contributions of SA, EG and APP, and their synergistic mechanisms in modifying RPUF can be summarized as presented in Table 4. This is manifested through the following:

(a) APP demonstrates reduced high-temperature heat release characteristics along with a certain degree of mechanical enhancement. However, it exhibits elevated smoke generation and accelerates RPUF degradation at lower temperatures.
(b) EG exhibits lower smoke emission, higher residual char yield, and relatively lower heat release. Nonetheless, it has a higher thermal conductivity and larger particle size, leading to more pronounced disruption in mechanical properties of RPUFs.
(c) SA does not alter the fire-retardant, heat release, or smoke characteristics of RPUF, yet it plays a vital role in reducing thermal conductivity and enhancing mechanical performance. And it effectively compensates for the shortcomings of APP and EG.

In summary, in the ternary modified RPUF system of this study, the synergistic flame-retardant effect of EG and APP contributes to the excellent flame-retardant performance of RPUF. However, the accurate control of the ratio between the two is necessary to achieve an efficient synergistic flame-retardant effect. Excessive use of either one alone cannot achieve simultaneously effective suppression of heat release and smoke generation, leading to an insufficient flame-retardant rating. Furthermore, the significant drawback of achieving efficient flame retardancy is the considerable weakening of the thermal insulation and mechanical properties of RPUF. In contrast, the addition of 1wt.% nanoscale SA into RPUF not only effectively prevents excessive agglomeration under the lubricating and dispersing effect of polymers but also uniformly distributes within the cell walls of RPUF, providing a cost-effective reinforcement (Figure 9). This precisely compensates for the weakening effects of EG and APP on the mechanical and thermal properties of RPUF. Both excessive or insufficient amounts of SA will fail to provide effective compensation. Ultimately, we obtained a novel EG/APP/SA/RPUF composite material with thermal insulation performance comparable to pure RPUF, while achieving a significant increase in compressive strength and obtaining a V-0 flame-retardant rating. This highlights the effective ternary synergistic effect with commercially available cost-effective EG, APP, and SA. Notably, through the controlled usage of SA, EG, and APP ratios, the design concept of effectively regulating flame retardancy, insulation, and mechanical performance in polymeric foams is achievable. This approach also provides a novel and affordable strategy reference for designing applications with different performance levels.

## 4. Conclusions

To address the significance and challenge of the balancing of high-strength, low thermal conductivity, and high flame resistance to the building wall insulation materials. This study developed ternary synergistic modification RPUF composites via commercially cost-effective EG/APP/SA. It is concluded that the addition of 1 wt.% SA substantially improved the compressive strength of RPUF from 229 kPa to 396 kPa while reducing thermal conductivity from 24.4 mW/mK to 19.8 mW/mK. The finding on the role of SA provides a new perspective in the field of material functional modification improve while sacrificing the insulation and mechanical properties. Based on the strengthen effect of SA/RPUF, the further introduction of EG and APP exhibited both synergistic effect in the compressive strength and flame retardant. Specifically, the compressive strength increased by 88.2% and 8.8%, respectively, compared to neat RPUF and SA/RPUF, when 2 wt.% of EG and 6 wt.% of APP were simultaneously incorporated. The flame retardant of the 6EG/2APP/SA/RPUF achieving a V-0 flame retardant rating. The residual weight at 700 °C increased from 19.2 wt.% to 30.9 wt.%. Future research focused on improving the dispersion uniformity and interface bonding of SA, EG, and APP through material modification and particle size optimization to further enhance this ternary synergistic efficiency is worthy of further study. This novel strategy of achieving balanced properties through the synergistic modification of commercially cost-effective materials holds promise for applications in the architectural industry, contributing to reduced building energy consumption and enhanced fire safety.

## Figures and Tables

**Table 1:** The basic formulation of neat RPUF.

Materials	4110 (g)	MDI (g)	AK158 (g)	A33 (g)	H_2_O (g)	141b (g)
RPUF	100	130	6	0.8	2	10

**Table 3:** Flammability test and CCT data of EG/APP/SA/RPUFs.

Samples Units	TTI (s)	PHRR (kW/m^2^)	THR (MJ/m^2^)	TSP (m^2^)	MRC (wt.%)
RPUF	2	256	42.9	4.58	0.23
SA/RPUF	2	276	42.3	4.74	0.24
8EG/SA/RPUF	7	340	39.9	2.89	7.24
8APP/SA/RPUF	5	464	32.1	7.37	4.35
2EG/6APP/SA/RPUF	6	392	36.4	6.20	4.92
4EG/4APP/SA/RPUF	7	365	37.6	4.23	6.06
6EG/2APP/SA/RPUF	7	311	39.0	3.78	6.85

**Table 4:** Contributions of SA, EG, and APP on RPUF performance.

Materials	Desired RPUF Performance						
	Low Thermal Conductivity	High Strength	High Flame Retardancy				
			Low Heat Release		Low Smoke Production	High Residual Mass	High LOI
			<300 °C	>300 °C			
APP	/	/	- -	+ +	- -	+	+
EG	- -	- -	+	/	+ +	+ +	+
SA	+ +	+ +	/	/	/	/	/
EG/APP/SA	+	+	/	+	+	+ +	+ +

Note: “+” indicates a positive effect, “+ +” indicates a significantly positive effect, “/” indicates no effect or unclear effect, “-” indicates a negative effect, and “- -” indicates a significantly negative effect.

## Data Availability

Data is contained within the article.

## Supplementary Materials

The following supporting information can be downloaded at: https://www.mdpi.com/article/10.3390/polym16030330/s1, Figure S1: The FT-IR spectra of (a) SA, (b) EG, (c) APP, and (d) neat RPUF, SA/RPUF and EG or APP modified SA/RPUF; Figure S2: The XRD spectra of (a) SA, (b) EG, (c) APP, and (d) neat RPUF, SA/RPUF an EG or APP modified SA/RPUF; Figure S3: Morphology and size of (a) SA, (b) EG, and (c) APP raw materials; Figure S4: Digital photographs of (a) neat RPUF, (b) SA/RPUF, (c) 8EG/SA/RPUF, (d) 8APP/SA/RPUF, (e) 2EG/6APP/SA/RPUF, (f) 4EG/4APP/SA/RPUF, and (g) 6EG/2APP/SA/RPUF. References [69,74,75,76,77,78] are cited in the supplementary materials.
