# Supplementary material for: Balanced Thermal Insulation, Flame-Retardant and Mechanical Properties of PU Foam Constructed via Cost-Effective EG/APP/SA Ternary Synergistic Modification
Source: Polymers (Basel). 2024 Jan 25;16(3):330. doi: 10.3390/polym16030330 (PMC10857279; doi:10.3390/polym16030330)
Supplement: Supplementary file 1 [file polymers-16-00330-s001.zip › polymers-2819870-supplementary.pdf]

Supporting Information

Balanced thermal insulation, flame-retardant and mechanical properties of PU foam constructed via cost-effective EG/APP/SA ternary synergistic modification

Hongfu Li [1,2,*], Longtao Hou [1,3], Yunpeng Liu [1,2], Zhiyu Yao [2], Lixing Liang [4], Dangxin Tian [2,5], Chunhui Liu [2,5], Junqiang Xue [2,5], Linshan Zhan [2,5], Yongqi Liu [2,5], Zhilu Zhen [2,5] and Kangmin Niu [1]

[1] School of Materials Science and Engineering, University of Science and Technology Beijing, Beijing 100083, China;

[2] Hebei Construction Group Corporation Limited, Baoding 071051, China;

[3] Hangzhou Hikvision Digital Technology Co., Ltd., Hangzhou 310052, China;

[4] Microelectronics and Information Materials Research Center, Hangzhou Innovation Institute, Beihang University, Hangzhou 310053, China;

[5] Hebei Province Prefabricated Building Technology Innovation Center, Baoding 071051, China

* Correspondence: lihongfu@ustb.edu.cn

Chemical structure of EG/APP/SA/RPUFs

The FTIR spectra of the various samples are depicted in Figure S1. It can be observed that the FTIR spectra of all RPUF samples in Figure S1(d) exhibit highly similar curves, indicating minimal occurrence of significant chemical structural changes in the modified RPUF. However, it is noteworthy that the small peak at 2280 cm^{-1} (typical residual N=C=O stretching vibration) becomes more pronounced with the addition of SA, EG, and APP. This phenomenon can be attributed to two potential causes: (1) the presence of hydroxyl groups on the filler surfaces, which have the capacity to alter the isocyanate index (RNCO/OH) [1]; (2) interference arising from the flame-retardant additives, resulting in alterations in the rheological behavior of the reaction mixture or coupling between surface groups and isocyanates, polyols, etc. [2]. In fact, both of these possibilities have the potential to induce lower crosslinking densities and influence the cell morphology as well as the mechanical and thermal insulation properties of the RPUF composite materials, which will be further discussed in subsequent sections.

The XRD spectra of the various samples are presented in Figure S2. From Figure S2(d), it can be observed that the XRD spectrum of SA/RPUF exhibits similar curves to neat RPUF, as the XRD spectrum of SA demonstrates broad peaks characteristic of an amorphous phase (Figure S2(a)). However, in the XRD curves of 8EG/SA/RPUF and 8APP/SA/RPUF, some new diffraction peaks are observed in the RPUF composite materials. Specifically, in 8EG/SA/RPUF, a diffraction peak appears at $2\theta=26.23°$ corresponding to the (002) plane of EG, while in the XRD curve of 8APP/SA/RPUF, characteristic peaks of APP can also be observed. These newly appearing peaks confirm the successful introduction of EG and APP.

More detailed functional groups or crystal planes corresponding to the characteristic peaks in

FTIR and XRD can be found in references [3-6].

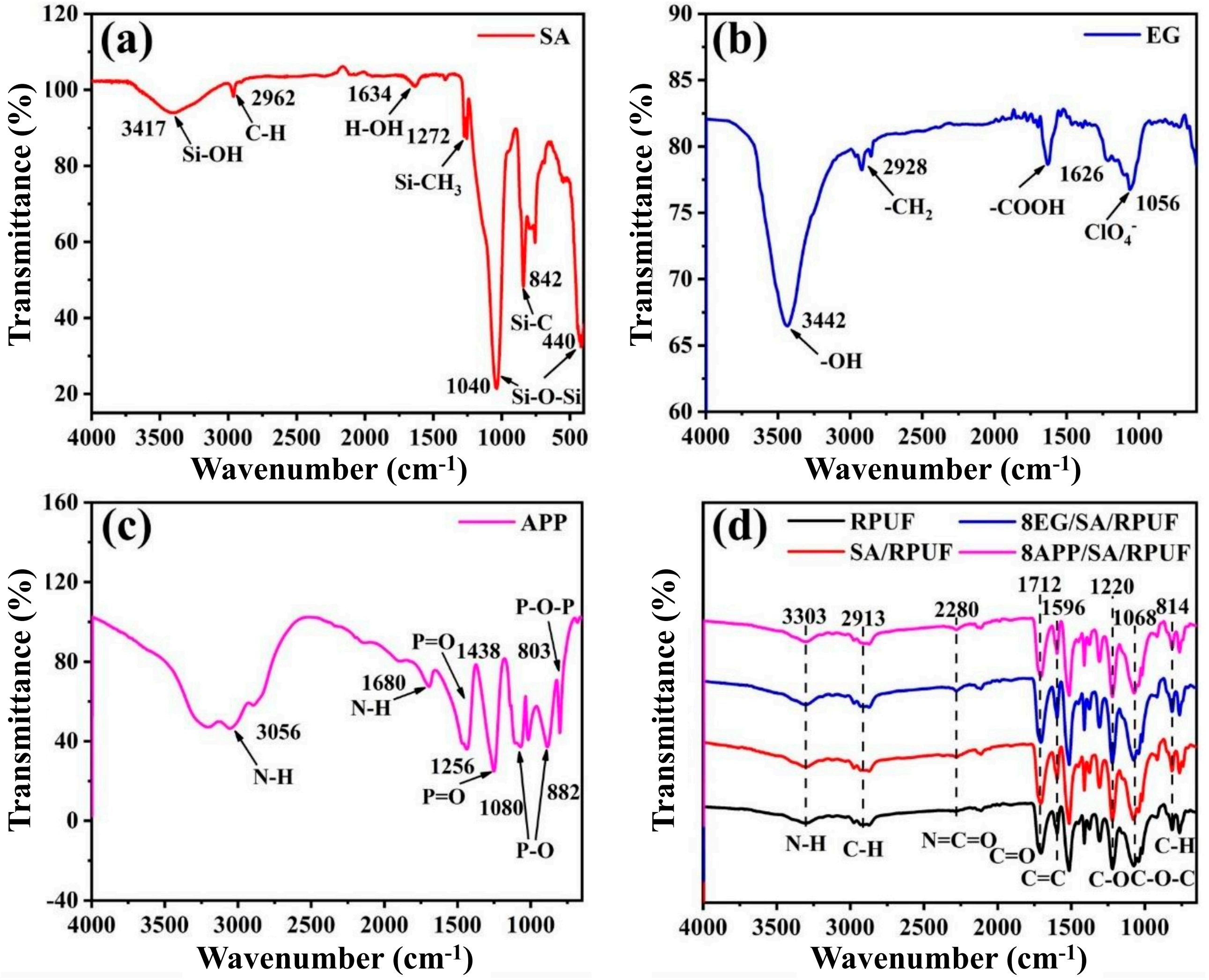

Figure S1. The FT-IR spectra of（a）SA,（b）EG,（c）APP, and（d）neat RPUF, SA/RPUF and EG or APP modified SA/RPUF

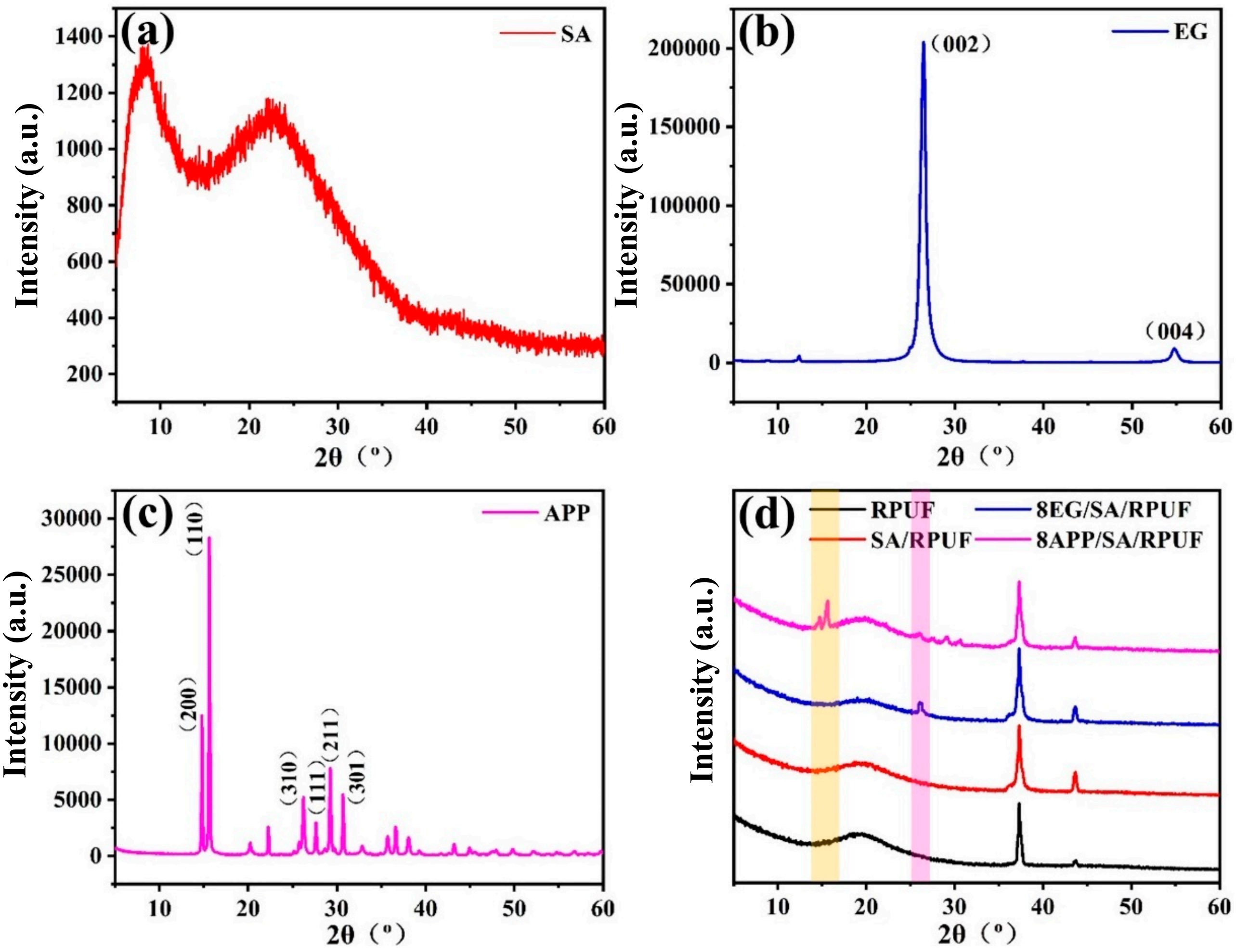

Figure S2. The XRD spectra of（a）SA,（b）EG,（c）APP, and（d）neat RPUF, SA/RPUF an EG or APP modified SA/RPUF

Microstructure of SA、EG、APP

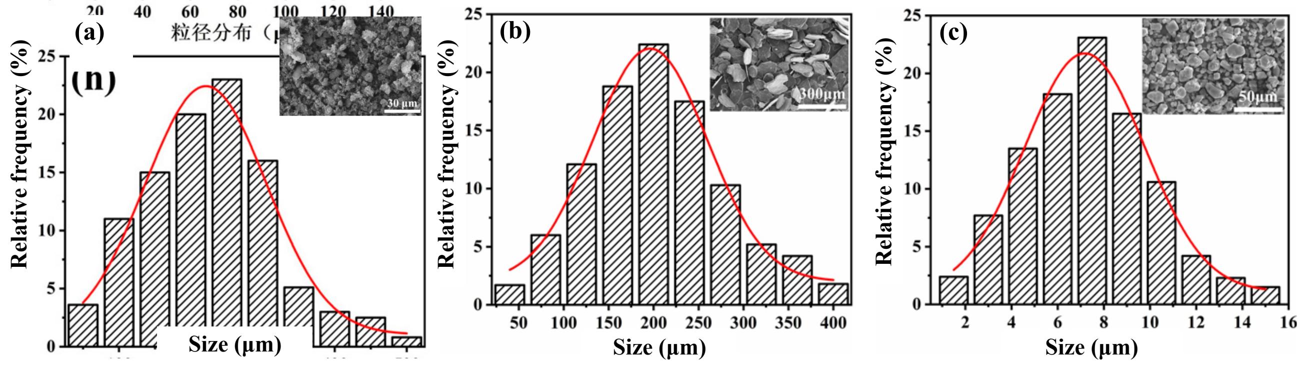

Figure S3. Morphology and size of (a) SA, (b) EG, and (c) APP raw materials

Microstructure of EG/APP/SA/PRUFs

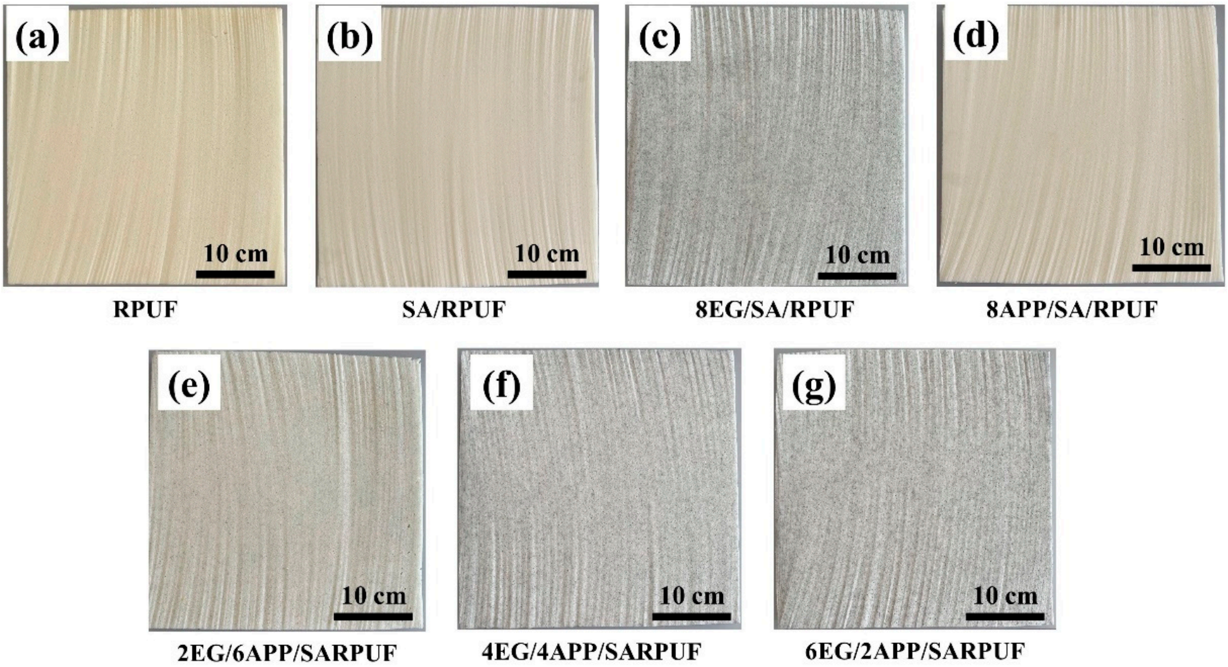

Figure S4. Digital photographs of (a) neat RPUF, (b) SA/RPUF, (c) 8EG/SA/RPUF, (d) 8APP/SA/RPUF, (e) 2EG/6APP/SA/RPUF, (f) 4EG/4APP/SA/RPUF, and (g) 6EG/2APP/SA/RPUF

References

[1] N. Gama, L.C. Costa, V. Amaral, A. Ferreira, A. Barros-Timmons, Insights into the physical properties of biobased polyurethane/expanded graphite composite foams, Compos. Sci. Technol. 138 (2017) 24-31.

[2] G. Harikrishnan, S.N. Singh, E. Kiesel, C.W. Macosko, Nanodispersions of carbon nanofiber for polyurethane foaming, Polymer 51(15) (2010) 3349-3353.

[3] M.Y. Shen, W.J. Chen, C.F. Kuan, H.C. Kuan, J.M. Yang, C.L. Chiang, Preparation, characterization of microencapsulated ammonium polyphosphate and its flame retardancy in polyurethane composites, Mater. Chem. Phys. 173 (2016) 205-212.

[4] B.B. Wang, X.D. Qian, Y.Q. Shi, B. Yu, N.N. Hong, L. Song, Y. Hu, Cyclodextrin microencapsulated ammonium polyphosphate: Preparation and its performance on the thermal, flame retardancy and mechanical properties of ethylene vinyl acetate copolymer, Compos. Part. B-Eng. 69 (2015) 22-30.

[5] X.Y. Pang, Y.P. Xin, X.Z. Shi, J.Z. Xu, Effect of different size-modified expandable graphite and ammonium polyphosphate on the flame retardancy, thermal stability, physical, and mechanical properties of rigid polyurethane foam, Polym. Eng. Sci. 59(7) (2019) 1381-1394.

[6] S.C. Huang, C. Deng, S.X. Wang, W.C. Wei, H. Chen, Y.Z. Wang, Electrostatic action induced interfacial accumulation of layered double hydroxides towards highly efficient flame retardance and mechanical enhancement of thermoplastic polyurethane/ammonium polyphosphate, Polym. Degrad. Stab. 165 (2019) 126-136.
